# Identifying oral disease variables associated with pneumonia emergence by application of machine learning to integrated medical and dental big data to inform eHealth approaches

**DOI:** 10.3389/fdmed.2022.1005140

**Published:** 2022-09-22

**Authors:** Neel Shimpi, Ingrid Glurich, Aloksagar Panny, Harshad Hegde, Frank A. Scannapieco, Amit Acharya

**Affiliations:** 1 Center for Clinical Epidemiology and Population Health, Marshfield Clinic Research Institute, Marshfield, WI, United States; 2 Cancer Care and Research Center, Marshfield Clinic Research Institute, Marshfield, WI, United States; 3 Security Health Plan, Marshfield Clinic Health System, Marshfield, WI, United States; 4 Berkeley Bioinformatics Open-source Projects, Lawrence Berkeley National Laboratory, Berkeley, CA, United States; 5 Department of Oral Biology, School of Dental Medicine, University at Buffalo, Buffalo, NY, United States; 6 Advocate Aurora Research Institute, Advocate Aurora Health, Chicago, IL, United States

**Keywords:** data mining, decision support tools, eHealth, health information system (HIS), electronic health records (EHR), information storage and retrieval, pneumonia, medical-dental integration

## Abstract

**Background:**

The objective of this study was to build models that define variables contributing to pneumonia risk by applying supervised Machine Learning-(ML) to medical and oral disease data to define key risk variables contributing to pneumonia emergence for any pneumonia/pneumonia subtypes.

**Methods:**

Retrospective medical and dental data were retrieved from Marshfield Clinic Health System’s data warehouse and integrated electronic medical-dental health records (iEHR). Retrieved data were pre-processed prior to conducting analyses and included matching of cases to controls by (a) race/ethnicity and (b) 1:1 Case: Control ratio. Variables with >30% missing data were excluded from analysis. Datasets were divided into four subsets: (1) All Pneumonia (all cases and controls); (2) community (CAP)/healthcare associated (HCAP) pneumonias; (3) ventilator-associated (VAP)/hospital-acquired (HAP) pneumonias and (4) aspiration pneumonia (AP). Performance of five algorithms were compared across the four subsets: Naïve Bayes, Logistic Regression, Support Vector Machine (SVM), Multi-Layer Perceptron (MLP) and Random Forests. Feature (input variables) selection and ten-fold cross validation was performed on all the datasets. An evaluation set (10%) was extracted from the subsets for further validation. Model performance was evaluated in terms of total accuracy, sensitivity, specificity, F-measure, Mathews-correlation-coefficient and area under receiver operating characteristic curve (AUC).

**Results:**

In total, 6,034 records (cases and controls) met eligibility for inclusion in the main dataset. After feature selection, the variables retained in the subsets were: All Pneumonia (*n* = 29 variables), CAP-HCAP (*n* = 26 variables); VAP-HAP (*n* = 40 variables) and AP (*n* = 37 variables), respectively. Variables retained (*n* = 22) were common across all four pneumonia subsets. Of these, the number of missing teeth, periodontal status, periodontal pocket depth more than 5 mm and number of restored teeth contributed to all the subsets and were retained in the model. MLP outperformed other predictive models for All Pneumonia, CAP-HCAP and AP subsets, while SVM outperformed other models in VAP-HAP subset.

**Conclusion:**

This study validates previously described associations between poor oral health and pneumonia. Benefits of an integrated medical-dental record and care delivery environment for modeling pneumonia risk are highlighted. Based on findings, risk score development could inform referrals and follow-up in integrated healthcare delivery environment and coordinated patient management.

## Introduction

Pneumonia continues to represent a significant medical condition associated with substantially increased morbidity, mortality and healthcare cost, especially with advancing age. The American Lung Association defines pneumonia as a common infection of the lung caused by bacteria, fungi and/or viruses (1). Treatment and management varies depending on the cause of pneumonia and symptom severity. In 2017, the National Hospital Ambulatory Medical Care Survey reported approximately 1.3 million visits to emergency departments with a primary diagnosis of pneumonia (2) and the CDC reported 49,157 deaths due to pneumonia in the same year (3). Pneumonia has five subtypes: aspiration pneumonia (AP), community-acquired pneumonia (CAP), hospital-acquired pneumonia (HAP), health care-acquired pneumonia (HCAP) and ventilator-associated pneumonia (VAP) (4, 5). In the timespan between 2010 and 2014, Corrado et al. reported that CAP is the most frequent subtype (54.3%) while VAP (1.6%) is the least frequent subtypes (6).

The current evidence base supports poor oral health as a risk factor for VAP and AP. In contrast with the now well-studied association between oral health and HAP, CAP remains underexplored by population-level studies (7). Notably, a 2017 systematic review of risk factors for adult CAP identified poor oral/dental health, including periodontal disease (PD) as potential risk factors (8, 9). However, a large population based study did not find PD to be a risk factor but did find dental caries and tooth loss to be associated with pneumonia (7). Notably, difficulty in defining the causal organism(s) in over 60% of pneumonia cases may be partially attributable to pathogenic emergence of normal oral flora (10), including anaerobes (11), consequential to environmental perturbation, indicating that some pneumonia may originate from oral dysbiosis (11). Viral infection may also represent a potential cause of infection unrelated to oral health status (12, 13). Moreover, poor oral health leads to a more anaerobic environment, which may contribute organisms that colonize both lungs and the oral cavity (14). Growing evidence supports the potential role of oral flora in the etiology of pneumonia (15–18). Comparison of pulmonary microbiota of patients admitted to the ICU with CAP, VAP and other HAP, in the context VAP (15), demonstrates clear overlaps across both microbiomes. A systematic review supported a 40% reduction in HAP incidence following improvement of oral hygiene in the hospital setting (19). By contrast, a clinical trial implementing improved oral hygiene in a nursing home setting was terminated early due to futility (20), so further study is warranted.

Currently, trends to transform care delivery across the siloed medical-dental domains include development of integrated patient-centric care delivery models. This has been supported by application of Artificial Intelligence (AI) in healthcare to develop translational e-Health approaches to facilitate implementation of precision care delivery (21–23). For example, machine learning (ML), sub-domain of AI, involves development of algorithms and make decisions or predictions relative to future data based on iterative modeling of historic patient data (21). Algorithms developed by these models can be translated at point of care in form of clinical decision support tools or risk prediction models (24–26).

Secondary use of electronically collected medical and dental data for elucidating associations between oral-systemic health conditions may expand insights into potential risk factors that contribute to pneumonia emergence in the context of the various pneumonia subtypes. Such characterization will contribute to development of eHealth approaches in emerging integrated medical and dental care delivery models. Because poor oral health is *a modifiable risk factor*, targeting oral disease prevention and treatment in the general population and high-risk sub-populations could help reduce pneumonia risk (27, 28). The objective of this study was to build models that define variables contributing to pneumonia risk by applying supervised Machine Learning (ML) to medical and oral disease data to define key risk variables contributing to pneumonia emergence for any pneumonia/pneumonia subtypes.

## Methods

### Study setting

This study was conducted at Marshfield Clinic Health System (MCHS) (28), a large multi-specialty healthcare practice with an expansive service area spanning largely rural tracts of central, western and northern Wisconsin. MCHS has partnered with the Family Health Center of Marshfield (FHC-M), whose service area largely overlaps that of MCHS (30). Medical and oral healthcare are delivered across the service area *via* a network of over 50 clinics and hospitals supported by an integrated medical-dental electronic health record (iEHR) that captures healthcare encounter data in real time. Data are backed up daily in the MCHS enterprise data warehouse (EDW), making this repository and iEHR among the largest combined medical and dental record systems in the country.

This study was reviewed and approved by the Institutional Review Board (IRB) of Marshfield Clinic Research Institute.

### Definition of the subject eligibility criteria

From a cohort of patients with medical and dental data, the following inclusion/exclusion criteria were applied:

- Patients >/= 21 years of age
- Patients with at least one oral examination at the FHC dental center between 2007 and 2019.
- Patients with at least two ambulatory visits within 3 years of their latest medical visit.
- Recurrent pneumonia episodes were excluded. Pneumonia recurrence was identified by documenting (additional ICD9/10 CM pneumonia associated codes) less than 90 days of the index pneumonia diagnosis.

Eligible patients (*n* = 6,034) were assigned to one of the two cohorts, based on their alignment with the following inclusion criteria:

Cases: were defined as patients who had a documented evidence of ICD9 CM (480.0–497.0) or ICD10 CM (J12-J18.9) and a pneumonia encounter between 01/01/2007 and 12/30/2019 as previously defined (31):

- Rule of one: pneumonia encounter with documented by ICD9/10CM codes and associated antibiotics prescription and/or a chest radiograph collected within ±30 days of the index pneumonia encounter.
- Rule of two: two pneumonia encounters in patients with documented by ICD9/10 CM codes during a pneumonia episode.

There was no overlap between “a” and “b”. Patients who did not meet the above definitions were excluded from further analyses.

Controls: Patients with no ICD9/10 CM code for pneumonia were included in the dataset.

### Data retrieval

To achieve the objective of the study, retrospective data from 2007 to 2019 were extracted from the MCHS EDW. A comprehensive list of potential data features were shortlisted following comprehensive review of prior studies that defined risk variables associated with definition of pneumonia and its various subtypes. Candidate potential risk factors targeted for further analysis are catalogued in Supplementary Table S1. The goal of predicting Pneumonia subtype risk was treated as a classification problem, stratifying patients who had a pneumonia diagnosis (cases) as “high risk” and those with no pneumonia diagnosis (controls) as “low risk”.

### Data preparation

Retrieved data were pre-processed prior to conducting analyses and included matching of cases to controls by (a) race/ethnicity and (b) 1:1 Case: Control ratio (24). Variables with >30% missing data were excluded from analysis. Datasets were divided into four subsets: (1) All Pneumonia (all cases and controls); (2) community (CAP) and healthcare associated (HCAP) pneumonias; (3) ventilator-associated (VAP) or hospital-acquired (HAP) pneumonias and (4) aspiration pneumonia (AP). An evaluation set (10%) was extracted from the subsets for further validation. There was no overlap among subtypes based on sub-classification process. The data were exhaustively classified using a rule based algorithm that examined each case for carefully defined sub-type specific features that would allow for unequivocal assignment to a specific subtype (31). Moreover, only validated pneumonia applying NLP to radiographic notes was included in the dataset (32).

### Feature selection

To identify a representative subset of attributes, a univariate filter, i.e., information gain with the ranker method was employed (33). Feature selection was conducted on all the four datasets using WEKA^®^ (34). This allowed for evaluating contribution of each variable by measuring the information gain with respect to the class, using the following formula:

(1.1) InfoGain(Class, Variable)=E(Class)−E(Class∣Variable)

where *E*, stands for entropy, which is defined as:

(1.2) E=−∑(Probabilityclass∗log2(Probabilityclass))

We set a cut-off value of ~3*10^−3^ as low information gain and variables with less than this value were further excluded from the analyses.

### Machine learning algorithms

Performance of five algorithms were compared across the four subsets: Naïve Bayes (NB), Logistic Regression (LR), Support Vector Machine (SVM), Multi-Layer Perceptron (MLP) and Random Forests (RF). Feature (input variables) selection and ten-fold cross validation was performed on all the datasets. Model performance was evaluated in terms of total accuracy, sensitivity, specificity, F-measure, Mathews-correlation-coefficient and area under receiver operating characteristic curve (AUC). Performance estimation was conducted using a stratified 10-fold cross validation approach. Table 1 summarizes the list of all data features included in the prediction model. The study utilized the implementation of these ML algorithms available in the Waikato Environment for Knowledge Analyses (WEKA) open source tool (34). ML was applied to rigorously processed datasets that included only validated pneumonia cases subtyped using a rule-based algorithm to prevent misclassification error.

### Performance measures

To assess the prediction model performance of difference algorithms, the study compared ML algorithms using the following performance measures.

1. The Area under the ROC curve (AUC) as defined by Hand and Till for binary classification (35).
(1.3) $$\text{AUC}=\frac{\frac{\left[S_{0}-n_{0}\left(n_{0}+1\right)\right]}{2}}{n_{0}n_{1}}$$
  where *n*_0_ and *n*_1_ are the numbers of “pneumonia cases” and “controls”, respectively, and $S_{0}=\sum r_{i}$, where *r*_*i*_ is the rank of the ith “pneumonia cases” in the ranked list.
2. Sensitivity, also termed recall, is the ratio of the number of correctly classified “pneumonia cases” instances to the total number of “controls” instances.
(1.4) $$\text{Recall}/\text{Sensitivity}(\text{Se})=\frac{TP}{TP+FN}$$
  where TP = true positive, FN = false negative.
3. Precision is the ratio of the number of correctly classified “pneumonia cases” instances to the total number of instances that are classified as “controls”.
(1.5) $$\text{Precision}=\frac{TP}{TP+FP}$$
  where FP = false positive.
4. Specificity is the ratio of the number of correctly classified “pneumonia cases” instances to the total number of instances that are classified as “controls”.
(1.6) $$\text{Specificity}=\frac{TN}{TN+FP}$$
  where TN = true negative.
5. Accuracy is the ratio of the number of correctly classified instances to the total number of instances.
(1.7) $$\text{Accuracy}=\frac{TN+TP}{TP+TN+FP+FN}$$
6. F-measure is the harmonic mean of precision and recall.
(1.8) $$\text{F}-\text{measure}=\frac{2*\;Precision\;*\;Recall}{Precision\;+\;Recall}$$
7. Matthew’s Correlation Coefficient (MCC) considers the accuracy and error rates and is calculated by the following equation:
(1.9) $$\text{MCC}=\frac{TP*TN*\;-FP*FN}{\sqrt{\left(TP+FP)(TP+FP)(TN+FP)(TN+FN\right)}}$$

## Results

In total, 6,034 records (equal number of cases and controls) met eligibility for inclusion in the main dataset. Table 1 shows the distribution of the datasets. A total of 52 variables which included demographic (*n* = 2), oral health (*n* = 6) and medical/environmental/behavioral (*n* = 44) were identified and retrieved from the data warehouse. Preprocessing of data resulted in deletion of three features (Arterial blood gas, Blood oxygen saturation levels and Pro-calcitonin levels) based on a high proportion of missing data. Among these (*n* = 49), a total of 43 variables (22 common and 21 unique variables) showed association with pneumonia. After performing feature selection on all 4 datasets, variables were excluded due to low information gains from the subsets respectively, thus bringing the variable countdown to: All Pneumonia (29 variables); CAP/HCAP (26 variables); VAP/HAP (40 variables) and AP (37 variables). Table 2 shows the variables retained in the different subsets. Variables retained (*n* = 22) were common across all four pneumonia subsets. The most significant feature in terms of information gain for dental variables included “restored teeth” (~0.3). Restored teeth was consistently the highest ranked variable across all pneumonia and pneumonia subtypes. While other dental variables such as missing teeth, periodontal pocket depth ≥5 mm, periodontal disease status and dentures were retained in all pneumonia models, they were differentially ranked with respect to level of contribution across the various pneumonia subtypes (see Table 2). Bleeding on probing was only retained in the AP model.

### Machine learning

**FIGURE 1:**
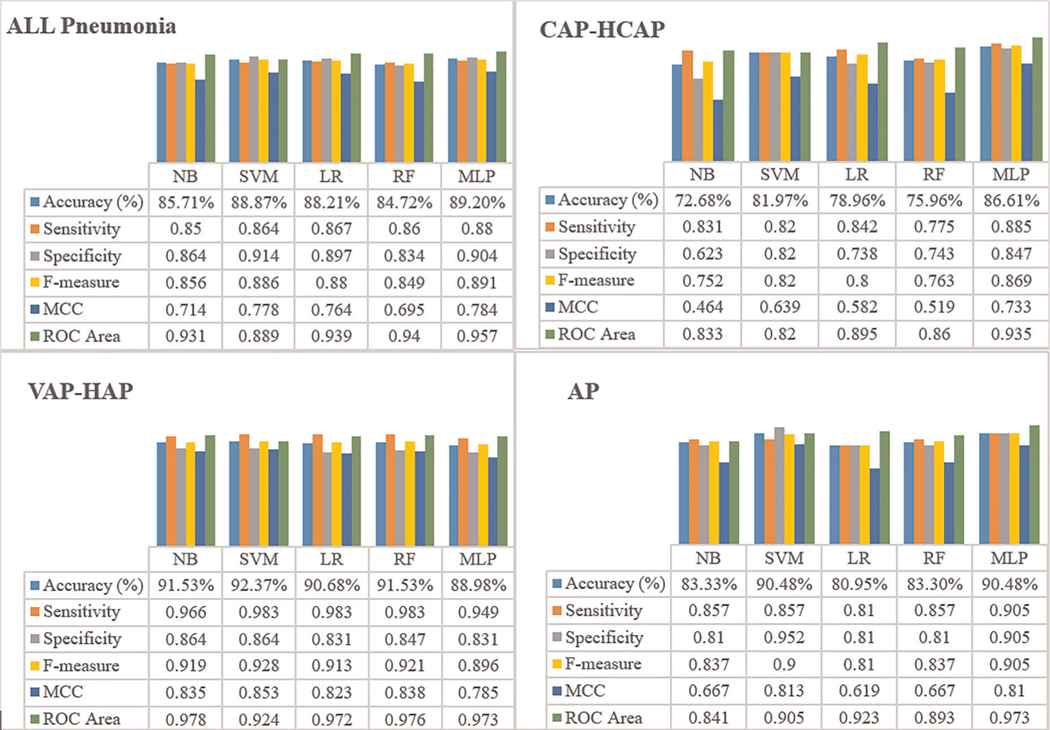
Results of the ML performance estimated through 10-fold cross validation.

Results of the performance estimated through 10-fold cross validation are shown in Figure 1. MLP demonstrated higher accuracy in classifying the patients with Pneumonia risk as compared to NB, LR, and SVM in all Pneumonia and CAP-HCAP subsets. In terms of sensitivity and specificity of the resultant models for all Pneumonia and CAP-HCAP subsets with the MLP algorithm demonstrated: all Pneumonia (sensitivity: 88% and specificity: 90%) and CAP-HCAP (sensitivity: 88% and specificity: 84%), respectively. By comparison, the sensitivity and specificity in VAP and AP subsets were: VAP [SVM, (sensitivity: 98%, specificity: 86%)] and AP [SVM, (sensitivity: 85%, specificity: 95%)] and [MLP, (sensitivity: 90.5%, specificity: 90.5%)], respectively.

**FIGURE 2:**
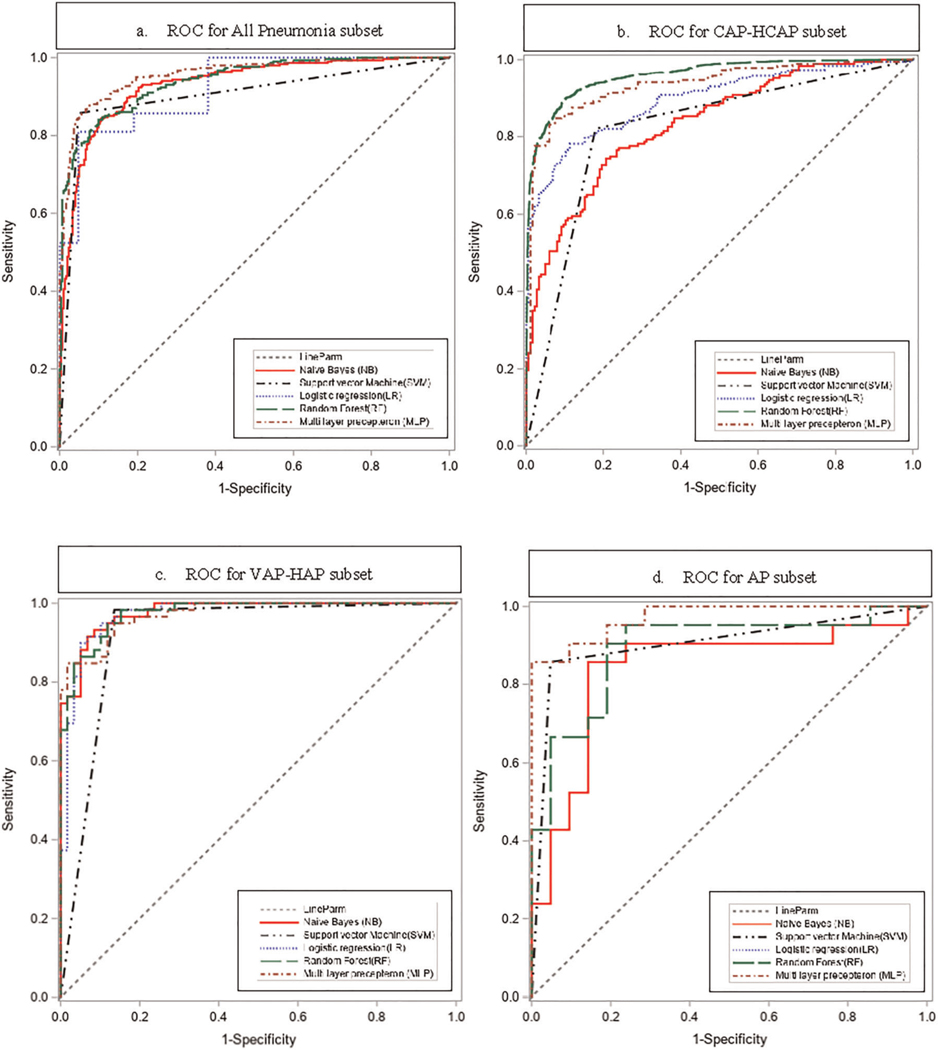
Receiver operating characteristics curve (ROC) for all pneumonia sub types.

The ROC curves are shown in Figure 2.

MLP outperformed other predictive models for All Pneumonia, CAP-HCAP and AP subsets, while SVM outperformed other models in VAP-HAP subset.

## Discussion

This study capitalized on the availability of rich, clinical real-world, population-based, “big data” available from an integrated medical-dental record and care delivery environment for modeling pneumonia risk through application of ML. Validated ML analytical approaches were applied to population-level data to vet association of established pneumonia risk factors including oral/dental variables with incidence of any pneumonia, as well as pneumonia subtypes including “CAP-HCAP”, “VAP-HAP” and “AP”. Among the ML algorithms used in this study, MLP yielded the best AUC (0.9) in “ALL pneumonia”, “CAP-HCAP” and “AP” subsets. Although predictive modelling using ML approaches have been used for various health conditions, use of ML approaches to define variables most predictive of pneumonia risk has been limited.

Notably, oral health-related variables defined in the current study that contributed most significantly to pneumonia risk are consistent with outcomes of oral diseases associated with infectious/inflammatory etiologies. Increasingly, a growing body of evidence supports plausibility of microbial pathogenesis as an important contributory factor underlying the association between pneumonia and oral diseases. Distinct pulmonary microbiomes in the upper and lower airways have recently been reported (11, 14). Notably, resident airway flora reflects microbiota found in the oral cavity, likely due to close proximity and interconnections between the lung and oral cavity (11). Further, shifts in microbial representation associated with disease processes such as cariogenesis and PD may also cause shifts in relative representation of oral microbiota in the pulmonary microbiomes (14). Such shifts in the relative representation of microbial species in the oral cavity in the context of infectious/inflammatory processes elicited by periodontal or cariogenic pathogens can lead to dysbiosis. Dysbiosis is associated with perturbation of the microbial content and environment giving rise to conditions unfavorable for normal flora which normally maintain microbial balance and the microbial environment. Subsequently, shifts in microbial representation may favor oral pathogens and establishment of conditions favorable to colonization by potential pulmonary pathogens. This positions these organisms to become opportunistic pathogens when conditions become favorable, especially in immunocompromised hosts. Moreover, direct transfer of oral bacteria from the oral cavity to the lungs may occur in the context of aspiration and VAP subtypes. Causality of VAP in conjunction with microbial transfer from the oral cavity during intubation has been definitively established by demonstrating genetic identity between the isolated pneumonia pathogen and bacterial isolates from dental plaque of the affected patient (16).

This study builds on two previous studies that applied informatics approaches to achieve pneumonia sub-classification into CAP, HCAP, VAP, HAP and ASP pneumonia subtypes in the same population analyzed in the current study (31, 32). We performed pneumonia case validation by using Natural Language Processing (NLP) on the observations recorded by radiologists on chest radiographs (31). The rules followed were based on the study published by Dublin et al. (36). A NLP based software was developed which enforced the rule-set prescribed by Dublin et al. and used to classify radiological records to have “positive”, “negative” or “unknown” mentions of pneumonia. This validated the presence of the pneumonia diagnoses in patients through unstructured data in addition to “Rule of one” and “Rule of two”. The validated pneumonia episodes after case validation were then classified into six cohorts. After extensive literature review, key medical features that were identified to classify pneumonia episodes and rules were set in place to develop a rule-based algorithm which allowed for classification of pneumonia episodes (32). Application of ML to data sets from our population-based pneumonia cohort that had previously undergone algorithm-driven sub-classification and validation of pneumonia status, ensured that all pneumonia sub classification assigned in the current study were accurate and represented true, validated pneumonia cases. Following propensity score adjustment for potential confounding by other established pneumonia risk factors, our group also conducted time to event analysis and statistical modeling in the same dataset. This alternative approach to exploring association between oral health status preceding pneumonia events similarly identified “missing teeth” and “periodontal status assigned by a dental professional” as two variables that were retained in statistical models as significant independent risk factors for pneumonia emergence.

Historically, similar studies developed predictive models to predict pneumonia risk in patients with specific systemic conditions including schizophrenia (37), liver transplantation (38), and traumatic brain injury (39). Another study modeled risk factors for 30-day hospital readmission following incidence of pneumonia (40). A recent study (41) developed ML VAP risk prediction models using EHR data from adult ICU encounters (*n* = 524 positive VAP patients) during the patients’ hospital stay (41). The authors reported logistic regression as the best performing model followed by MLP. The AUC (ROC) reported by the investigators was 0.8 after reviewing 48 h of data (41). The performance for VAP/HAP subset in our study was 0.9 and is likely attributable to volume of data used (591 positive VAP patients), the number of variables used (*n* = 40) [vs. 10 variables modeled in the study (41)], and use of 10% vs. 20% evaluation set in their study. Similarly, Xu et al. built models to predict adverse outcomes in patients with CAP using nine ML algorithms and reported AUC of 0.8 using MLP for prediction of death in pneumonia patients (42). The study reported using variables including fever, cough, tachypnea, dyspnea, hypertension, hematocrit, hemoglobin, WBC, creatinine, BUN, glucose, heart disease, immunosuppression, malignancy, cerebrovascular disease, renal disease and liver disease, which were also retained in our models using feature selection (42).

Our study focused on improving overall predictive accuracy by including medical and dental variables to develop a risk model for assessing patient risk for pneumonia. This study demonstrated that dental variables such as restored teeth, missing teeth, periodontal pocket depth ≥5 mm, periodontal disease status assigned by dental provider and presence of dentures, displayed high predictive performance. Selection of dental data variables also led to a novel observation: tooth restoration history and missing teeth play a significant role in pneumonia risk. Increased numbers of restorations and/or missing teeth may be related to higher risk of aspiration and perhaps dysphagia, well-known risk factors for pneumonia (43). These findings are similar to a recent population-based study conducted by Son et al. who showed that the risk of pneumonia significantly increased in patients with higher number of dental caries and missing teeth. In the present study, PPD ≥ 5 mm and periodontal status assigned by a dental provider were identified as significant factors contributing to pneumonia risk in all subsets. This observation further reinforces association between periodontal disease and pneumonia risk as shown in other studies (8, 10). Further in our study, “restored teeth” was the dental variable contributing to highest pneumonia risk. Two additional studies that applied ML to evaluate potential risk variables associated with HAP (37) and VAP (39), identified WBC count and serum sodium levels as additional crucial risk factors in their respective studies. WBC count was also identified as a significant variable in the current study. A recent study by Zhao et el. applied ML approaches to datasets of pneumonia and COVID patients and similarly showed that clinical indicators including WBC count may be a significant factor to predict the disease progression and outcomes in patients with pneumonia and COVID-19 (44).

The study acknowledges some limitation. The study data used was collected from a single healthcare system. This may raise potential for selection bias within the healthcare system. Generalizability of the predictive models developed in this study will require further testing and validation in other healthcare systems. Some of variables such as clinical attachment loss and other variables were not included due to incomplete and missing data. The addition of these variables may be necessary to further improve the pneumonia risk predictability in healthcare settings.

## Conclusion

The results of this study show that ML approaches that include medical and dental data show an association of oral health variables with pneumonia subtypes. Thus, consideration of oral health outcomes in the integrated healthcare environment would improve patient care through early detection of pneumonia risk and further help in clinical decision support to undertake preventive approaches.

To the best of our knowledge, this study is the first to develop predictive models using ML techniques for identifying risk factors associated with emergence of different pneumonia subtypes based on modeling of medical and dental data. Risk scores could be developed to inform patient referral and follow-up in integrated medical-dental care delivery settings, and coordination of oral health and pneumonia management. Future studies would test portability and translation of e-Health approaches into clinical care delivery in diverse healthcare settings.

## Supplementary Material

Supplementary Table 1: Candidate potential risk factors targeted for data analysis

## Figures and Tables

**TABLE 1:** Distribution of data across all pneumonia and subtypes.

Subsets	Cases (*n*)	Controls (*n*)	Total (*n*)	“*n*” for training	“*n*” for validation

All pneumonia	3,017	3,017	6,034	5,432	602
CAP/HCAP	1,832	1,832	3,664	3,298	366
VAP/HAP	591	591	1,182	1,064	118
AP	213	213	426	384	42

**TABLE 2:** Summarizes the list of all data features included in the prediction model.

	All pneumonia	CAP-HCAP	VAP-HAP	AP

1	Restored teeth	Restored teeth	Restored teeth	Restored teeth
2	Cough	Cough	Complete blood count	Dysphagia
3	Intubation	Intubation	White blood cell count	Video fluoroscopy
4	Complete blood count	Age	Heart failure	Age
5	White blood cell count	Complete blood count	Hematocrit	Missing teeth
6	Heart failure	White blood cell count	C-Reactive protein	Hematocrit
7	Hematocrit	Fever	Legionella urinary antigen test (ULA)	Complete blood count
8	Missing teeth	Beta lactam medication	Renal disease	Heart failure
9	Fever	Dyspnea	Blood urea nitrogen	White blood cell count
10	Blood urea nitrogen	Missing teeth	Dyspnea	Blood urea nitrogen
11	Dyspnea	Periodontal disease status	Cough	Renal disease
12	Age	Legionella urinary antigen test (ULA)	Blood glucose	Intubation
13	Beta lactam medication	Hematocrit	Intubation	Cerebrovascular disease
14	C-Reactive protein	Periodontal pocket depth >5mm	Missing teeth	Neoplastic disease
15	Legionella urinary antigen test (ULA)	Dentures	Fever	Dyspnea
16	Blood glucose	Heart failure	Sodium levels	Legionella urinary antigen test (ULA)
17	Renal disease	Blood urea nitrogen	Diabetes	Hypertension
18	Diabetes	Gender	S. Pneumoniae urinary antigen test (UAT)	S. Pneumoniae urinary antigen test (UAT)
19	Periodontal disease status	C-Reactive protein	Periodontal pocket depth >5mm	Periodontal disease status
20	Video fluoroscopy	Hypertension	Age	Bleeding on Probing
21	Sodium levels	Glucose	Video fluoroscopy	Blood glucose
22	S. Pneumoniae urinary antigen test (UAT)	Blastomycosis	Beta lactam medication	Steroid medication
23	Dysphagia	S. Pneumoniae urinary antigen test (UAT)	Periodontal disease status	Cryptococcosis
24	Periodontal pocket depth >5mm	Diabetes	Blastomycosis	Betalactam medication
25	Cerebrovascular disease	Video fluoroscopy	Cerebrovascular disease	Hypercholesterolemia
26	Blastomycosis	Hypercholesterolemia	Hypercholesterolemia	Aminoglycoside medication
27	Gender		Liver disease	Hemoglobin
28	Tachycardia		Tachycardia	Sodium levels
29	Histoplasmosis		Histoplasmosis	Tachypnea
30			BOP	Blastomycosis
31			Neoplastic disease	Fever
32			Hypotension	Gender
33			Haemoglobin	Histoplasmosis
34			Gender	Hypotension
35			Steriod medication	Periodontal pocket depth >5mm
36			Bradypnea	Diabetes
37			Hypertension	Dentures
38			Nausea	
39			Tachypnea	
40			Dysphagia	

Variables that were not retained included: chills, chest sounds, confusion, malaise, carbapenam and cephalosporins.

## Data Availability

The datasets presented in this article are not readily available because of private clinical data and is owned by Marshfield Clinic and is not available for public sharing. Requests to access the datasets should be directed to amit.acharya@aah.org.
