# Supplementary material for: Identifying oral disease variables associated with pneumonia emergence by application of machine learning to integrated medical and dental big data to inform eHealth approaches
Source: Front Dent Med. Author manuscript; Available in PMC 2023 Jan 12. (PMC9835559; doi:10.3389/fdmed.2022.1005140)
Supplement: Supplementary Table 1: Candidate potential risk factors targeted for data analysis [file NIHMS1840741-supplement-Supplementary_Table_1___Candidate_potential_risk_factors_targeted_for_data_analysis.docx]

**Supplementary Table 1:** Candidate potential risk factors targeted for data analysis

1. **Medical variables**

| **Type** | **No** | **Variable** | **Definition and documentation in EHR** | **Value** |
| --- | --- | --- | --- | --- |
| Demographics | 1. | Age | >21 years | 21-30; 31-40, 41-50, 51-60, 61-70, 71-80, 81-90, 90+ |
|  | 2. | Gender | Documented in EHR | Female; Male |
| Symptomology | 3. | Bradypnea | Defined as respiratory rate of <13rpms or documentation of ICD10CM: R06.89 | 1=Yes; 0=No |
|  | 4. | Chest sounds | ICD9CM:786.7; ICD10CM:R09.89 | 1=Yes; 0=No |
|  | 5. | Chills | ICD9CM:780.64; ICD10CM:R68.83 | 1=Yes; 0=No |
|  | 6. | Confusion | ICD9CM:780.97; ICD10CM:R41.82 | 1=Yes; 0=No |
|  | 7. | Cough | ICD9CM:786.2; ICD10CM:R05 | 1=Yes; 0=No |
|  | 8. | Dysphagia | ICD9CM:787.2; ICD10CM:R13.10 | 1=Yes; 0=No |
|  | 9. | Dyspnea | ICD9CM:786.0; ICD10CM:R06.9 | 1=Yes; 0=No |
|  | 10. | Fever | Defined as body temperature >100. 4°F or 38°C ICD9CM:780.60; ICD10CM:R50.9 | 1=Yes; 0=No |
|  | 11. | Hypotension | Defined as systolic blood pressure (bp) < 90mm of Hg and diastolic bp < 90mm of Hg  ICD9CM:458.9; ICD10CM:I95.9 | 1=Yes; 0=No |
|  | 12. | Hypothermia | Defined as body temperature <95°F or 35°C  ICD9CM:991.6; ICD10CM:R68.0 | 1=Yes; 0=No |
|  | 13. | Malaise | ICD9CM:780.79; ICD10CM:R53.8 | 1=Yes; 0=No |
|  | 14. | Nausea | ICD9CM:787.01; ICD10CM:R11.2 | 1=Yes; 0=No |
|  | 15. | Tachycardia | Defined as pulse >125 beats/min or  ICD9CM:785.0; ICD10CM: R00.0 | 1=Yes; 0=No |
| Laboratory values  Comorbidities | 16. | Arterial Blood Gas (ABG) | Defined as arterial pH <7.0 or  CPT code: 82803, 82805 | 1=Yes; 0=No |
|  | 17. | Arterial Oxygen Saturation | Defined as oxygen saturation levels <90% or arterial blood gas <60 mm of Hg or  CPT code: 82803, 94760 | 1=Yes; 0=No |
|  | 18. | Blastomycosis | CPT code: 86612 | 1=Yes; 0=No |
|  | 19. | Cryptococcicosis | CPT code: 86406 | 1=Yes; 0=No |
|  | 20. | Blood Glucose Levels | Defined as Hemoglobin A1C (HbA1C) >/=6.5% (48mmol/L) or Fasting Blood Glucose >/=126mg/dL (7mmol/L) or Random Blood Glucose >/=200mg/dL (11.1mmol/L) or CPT code: 82947 | 1=Yes; 0=No |
|  | 21. | Blood Urea Nitrogen (BUN) | Defined as urea more than >30mg/dL  CPT codes: 84520 , 80048 | 1=Yes; 0=No |
|  | 22. | Complete Blood Count (CBC) | Defined as Hemoglobin (Hb) <7.0 or Hematocrit value <30% or White Blood Cell count >10,000  CPT code: 85025 | 1=Yes; 0=No |
|  | 23. | C-Reactive Protein (CRP) | CPT code: 86140 | 1=Yes; 0=No |
|  | 24. | Hemoglobin (Hb) | Defined as Hb <7.0 ;CPT code: 83036 | 1=Yes; 0=No |
|  | 25. | Hematocrit | Defined as <30% ; CPT code: 85014 | 1=Yes; 0=No |
|  | 26. | Hypercholesterolemia | Total cholesterol >/=240 or High Density Lipids >/=60 or Low Density Lipids>/=160 or Triglycerides: >/=200 | 1=Yes; 0=No |
|  | 27. | Histoplasmosis | CPT code: 86698 | 1=Yes; 0=No |
|  | 28. | Pro calcitonin | Defined as 2-<10 or >10μg/L; CPT code: 84145 | 1=Yes; 0=No |
|  | 29. | Sodium levels | Defined as <130mEq/L; CPT code: 84295 | 1=Yes; 0=No |
|  | 30. | *S. Pneumoniae* urinary antigen test (UAT) | CPT code:183009 | 1=Yes; 0=No |
|  | 31. | Legionella urinary antigen test (ULA) | CPT code: 87449 | 1=Yes; 0=No |
|  | 32. | White Blood Cells (WBC) | Defined as WBD >10,000;  CPT code: 85048 | 1=Yes; 0=No |
| Comorbidities | 33. | Cerebrovascular disease | Documentation of ICD 9CM:430-437;  ICD10 CM: I60, I61, I662, I63, I65, I66, I67, I68, I69, | 1=Yes; 0=No |
|  | 34. | Diabetes | Documentation of ICD 9CM: 250(excludes codes for type 1), 250.0, 250.2-250.9, 250.02, 720.29; ICD10 CM:E11, E11.630, E11.64, E11.63, E11.638, E11.65, R73.03 | 1=Yes; 0=No |
|  | 35. | Heart failure | Documentation of ICD 9CM:428.1, 428.2, 428.20, 428.22, 428.23, 428.3, 428.30, 428.31, 428.32, 428.33, 428.4, 428.40, 428.41, 428.42, 428.43, 428.9; ICD10 CM:I50.2,I50.2, I50.20, I50.21, I50.22, I50.23, I50.3,I50.31, I50.32, I50.33, I50.4, I50.40, I50.41, I50.42, I50.43, I50.8, I50.81, I50.810, I50.811, I50.812, I50.813, I50.814, I50.82, I50.83, I50.84, I50.89, I50.9 | 1=Yes; 0=No |
|  | 36. | Hypertension | Grade 1: 140-159 systolic bp &/or 90-99 diastolic bp; Grade 2: 160-179 systolic bp &/or 100-109 diastolic bp; Grade 3 >/=180 systolic bp &/or >/=110 diastolic bp; | 1=Yes; 0=No |
|  | 37. | Chronic Kidney Disease | Documentation of ICD 9CM:585.1,585.2, 585.3, 585.4, 585.5, 585.6, 585.9; ICD10 CM:N18.1,N18.2, N18.3, N18.4, N18.5, N18.6, N18.9 | 1=Yes; 0=No |
|  | 38. | Liver disease | Documentation of ICD 9CM:571.0, 571.2, 571.4,571.5,571.6,571.8,571.9; ICD10 CM:K70, K71, K72, K73, K74, K75, K76, K77 | 1=Yes; 0=No |
|  | 39. | Neoplastic disease | Documentation of ICD 9CM:140-149, 150-159, 160-169, 170-175, 176, 179-189, 190-199, 200-208, 209, 210-229, 230-234, 235-238, 239; ICD10 CM:C15-C29, C30-C39, C40-C41, C43-44, C45-C49, C50-C58, C60-C63, C64-C68, C69-C72, C73-C75, C76-C80, C81-C96, C97, D00-D09, D10-D36, D37-D48 | 1=Yes; 0=No |
|  | 40. | Aminoglycosides | Amikacin, Gentamycin, Tobramycin, Linezolid, Vancomycin, or Colistin | 1=Yes; 0=No |
|  | 41. | Beta lactam | Ampicillin-Sublactam, or Piperacillin-Tazobactam | 1=Yes; 0=No |
|  | 42. | Carbapenem | Doripenam, Etrapenam, Imipenam-Cilastatin, or Meropenam | 1=Yes; 0=No |
|  | 43. | Cephalosporin | Cefptaxime, Cefepime, Ceftadizime, or Ceftriaxone | 1=Yes; 0=No |
|  | 44. | Steroid | Corticosteroids, Cortisones, Prednisone, methylprednisolonebetamethasone ,Dexamethasone, Hydrocortisone, Deflazacort (steriods prescribed for pneumonia) | 1=Yes; 0=No |
| Other | 45. | Video fluoroscopy | CPT code: 92611 | 1=Yes; 0=No |
|  | 46. | Intubation | CPT code: 31500 | 1=Yes; 0=No |

1. **Dental Variables**

| **Variables** |  | Cases(Definition) | Controls(Definition) | Values |
| --- | --- | --- | --- | --- |
| Periodontal status | 47. | All episodes with a value of periodontal status within 365 days before the pneumonia svc date (closest to the pneumonia svc date of an episode)(+)All episodes with a value of periodontal status between episodes start and episode end date (closest to the index date) | Latest periodontal status before the last dental visit. | Healthy  Type1  Type2  Type3  Type4  Type5 |
| PD>5mm | 48. | All episodes with a value for number of teeth with PD>=5mm within 365 days before the pneumonia svc date (closest to the pneumonia svc date of an episode)(+)All episodes with a value for number of teeth PD>=5mm between episodes start and episode end date (closest to the episode start date) | Latest value for "number of teeth PD>=5" before the last dental visit. | 0 teeth='0' 1-5 teeth ='1to5' 6-10 teeth='6to10' 11-15 teeth='11to15' 16-20 teeth ='16to20' 21-25 teeth='21to25' 26-28 teeth='26above' |
| Bleeding on probing ‘Yes’ | 49. | Number of teeth with BOP for all the pneumonia episodes were the value for variable "PD>5mm" is not missing. We imputed the value for "Bleeding YES" as zero if it is missing for an pneumonia episode where the value for "PDMoreThan5" is not missing. | Latest value for "number of teeth with BOP" before the last dental visit. |  |
| Restored teeth | 50. | The value for Number of teeth with "restorations" for all pneumonia episodes before an episode end date (closest to the episode start date) | Latest value for "number of teeth with restorations" before the last dental visit. |  |
| Missing teeth | 51. | The value for Number of teeth missing for all pneumonia episodes before an episode end date (closest to the episode start date) | Latest value for "number of missing teeth" before the last dental visit. |  |
| Dentures | 51. | The Presence or absence of a partial or complete denture for all pneumonia episodes before an episode end date | Latest value for "Presence or absence of a partial or complete denture" before the last dental visit | 1=Yes 0=No |
